# Dopamine D2 Receptors in the Paraventricular Thalamus Attenuate Cocaine Locomotor Sensitization

**DOI:** 10.1523/ENEURO.0227-17.2017

**Published:** 2017-10-24

**Authors:** Abigail M. Clark, Felix Leroy, Kelly M. Martyniuk, Wendy Feng, Erika McManus, Matthew R. Bailey, Jonathan A. Javitch, Peter D. Balsam, Christoph Kellendonk

**Affiliations:** 1 Graduate Program in Neurobiology and Behavior, College of Physicians and Surgeons, Columbia University, New York, NY 10032; 2 Department of Psychiatry, College of Physicians and Surgeons, Columbia University, New York, NY 10032; 3 Department of Pharmacology, College of Physicians and Surgeons, Columbia University, New York, NY 10032; 4 Department of Neuroscience, College of Physicians and Surgeons, Columbia University, New York, NY 10032; 5 Division of Molecular Therapeutics, New York State Psychiatric Institute, New York, NY 10032; 6 Department of Psychology, Barnard College Columbia University, New York, NY 10027

**Keywords:** anatomical tracing, cocaine sensitization, dopamine D2 receptors, fear conditioning, midline thalamus, paraventricular thalamus

## Abstract

Alterations in thalamic dopamine (DA) or DA D2 receptors (D2Rs) have been measured in drug addiction and schizophrenia, but the relevance of thalamic D2Rs for behavior is largely unknown. Using *in situ* hybridization and mice expressing green fluorescent protein (GFP) under the *Drd2* promoter, we found that D2R expression within the thalamus is enriched in the paraventricular nucleus (PVT) as well as in more ventral midline thalamic nuclei. Within the PVT, D2Rs are inhibitory as their activation inhibits neuronal action potentials in brain slices. Using Cre-dependent anterograde and retrograde viral tracers, we further determined that PVT neurons are reciprocally interconnected with multiple areas of the limbic system including the amygdala and the nucleus accumbens (NAc). Based on these anatomical findings, we analyzed the role of D2Rs in the PVT in behaviors that are supported by these areas and that also have relevance for schizophrenia and drug addiction. Male and female mice with selective overexpression of D2Rs in the PVT showed attenuated cocaine locomotor sensitization, whereas anxiety levels, fear conditioning, sensorimotor gating, and food-motivated behaviors were not affected. These findings suggest the importance of PVT inhibition by D2Rs in modulating the sensitivity to cocaine, a finding that may have novel implications for human drug use.

## Visual Abstract

**Figure:**
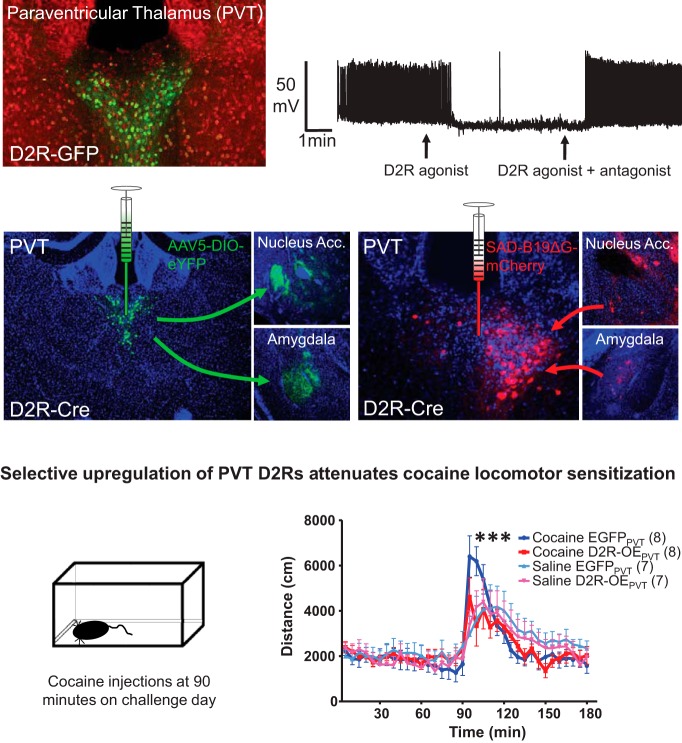

## Significance Statement

Alterations in thalamic dopamine (DA) or DA D2 receptors (D2Rs) have been measured in drug addiction and schizophrenia. However, although D2Rs have been extensively studied in the striatum, the relevance of thalamic D2Rs for neuronal function as well as behavior is largely unknown. Therefore, the significance of the human imaging findings for psychiatric disorders is unclear. Here, we found that the midline thalamus displays enriched expression of D2Rs whose activation inhibits thalamic neuron activity. Overexpression of D2Rs in the paraventricular nucleus (PVT), a dorsal midline thalamic nucleus, attenuated cocaine locomotor sensitization. This suggests that D2R-mediated inhibition of the PVT modulates the sensitivity to cocaine, a finding which has potential relevance for human drug use.

## Introduction

Historically, dopamine (DA) and its receptors have been most extensively studied in the dorsal and ventral striatum due to its strong dopaminergic innervation and high expression levels of DA receptors (Gerfen and Surmeier, 2011). In addition, DA neurons originating in the VTA innervate extrastriatal areas including the hippocampus, amygdala, and cortex (Yetnikoff et al., 2014). These projections have been well studied in motivated and cognitive behaviors, with dysfunction of these pathways implicated in schizophrenia and other mental disorders (Arnsten et al., 2015; Rosen et al., 2015; De Bundel et al., 2016).

However, much less is known about the functional significance of DA projections arising from the hypothalamus, periaqueductal gray (PAG), and locus coeruleus (LC). Toward this aim, recent work elucidated the importance of DA projections from the LC to the hippocampus in learning and memory (Kempadoo et al., 2016; Takeuchi et al., 2016). Similarly, studies in rats demonstrated that the paraventricular nucleus (PVT) of the thalamus receives innervation from DA neurons within the hypothalamus as well as the PAG, yet the function of this projection remains largely unknown (Li et al., 2014a).

In humans, PET imaging studies implicate a dysfunction of the striatal DA system in several neuropsychiatric and neurologic diseases including Parkinson’s disease, schizophrenia, and drug addiction (Albin et al., 1989; Abi-Dargham et al., 2000; Howes et al., 2012; Volkow and Morales, 2015). In contrast, the significance of abnormalities in extrastriatal DA systems in these disorders is unknown.

With the development of high affinity ligands for DA or DA D2 receptors (D2Rs), D2R density as well as psychostimulant-induced DA release can now be quantified in extrastriatal regions in the human brain (Kegeles et al., 2010). One extrastriatal region that has attracted attention is the thalamus, as both increased and decreased D2R levels have been observed in this region in patients with schizophrenia (Talvik et al., 2003; Yasuno et al., 2004; Buchsbaum et al., 2006; Talvik et al., 2006; Tuppurainen et al., 2006; Kessler et al., 2009; Kegeles et al., 2010; Seeman, 2013). Additionally, in cocaine addiction, psychostimulant-induced DA release is enhanced in the thalamus, and this release is associated with enhanced craving for cocaine as well as increased prefrontal metabolism (Volkow et al., 1997, 2005). However, despite these exciting clinical findings, little is known about the basic functions that are mediated by D2Rs in the thalamus. Moreover, human PET imaging studies have limited spatial resolution, thereby preventing the study of D2Rs in specific thalamic subnuclei.

Here, we take advantage of the ability to target specific brain circuits in the mouse to study the basic function of D2Rs in the thalamus. Using *in situ* hybridization and genetically modified mice that express green fluorescent protein (GFP) under the control of the *Drd2* receptor gene promotor, we first analyzed the expression pattern of thalamic D2Rs and show dense expression within the PVT as well as more ventral midline thalamic nuclei. Next, we recorded from GFP-expressing neurons in the PVT of Drd2-EGFP mice to determine the effect of PVT D2Rs on thalamic relay neuron activity. Using Cre-dependent anterograde and retrograde tracing methods, we further determined the brainwide connectivity pattern of D2R-expressing PVT neurons. Finally, we determined the behavioral significance of PVT D2Rs. to do this, we selectively overexpressed D2Rs in the PVT and tested these mice in a battery of behavioral tests that are supported by nucleus accumbens (NAc) and amygdala function and that also have relevance for the negative symptoms of schizophrenia and cocaine abuse in humans.

We found that D2Rs are most densely expressed in the midline thalamus and inhibit action potential firing of thalamic relay neurons. Additionally, we observed that D2R-expressing PVT neurons are part of a larger limbic circuit in the brain. Last, we identified a new role for PVT D2Rs in attenuating cocaine locomotor sensitization. These findings suggest that D2R-mediated inhibition of thalamic midline neurons modulates the sensitivity to cocaine, a finding that may have implications for human drug abuse.

## Materials and Methods

### Animals

Animals were housed with ad libitum access to food and water. For operant-based tasks and novelty suppressed feeding, mice were food restricted and maintained at 85% of baseline body weight. A 12/12 h light/dark schedule in a temperature and humidity controlled environment was maintained. Three mouse lines were used: wild-type C57BL/6J, as well as Drd2-Cre(ER44Gsat/Mmucd, RRID:MMRRC_032108-UCD) and Drd2-EGFP (S118Gsat/Mmnc, RRID:MMRRC_000230_UNC) both backcrossed onto a C57BL/6J background. All behavioral testing was performed during the light cycle. Experiments were approved by the Institutional Animal Care and Use Committee.

### Animals for behavioral testing

Cohort 1 underwent behavioral testing in the following order: elevated plus maze (EPM), open field (OF), light-dark (LD) test, prepulse inhibition (PPI), Pavlovian-to-instrumental transfer (PIT), progressive ratio (PR), devaluation fear conditioning, and cocaine sensitization. Cohort 2 underwent cocaine sensitization followed by fear conditioning. Both cohorts were analyzed postmortem for the viral expression pattern using immunohistochemistry (IHC). Cohorts 1 and 2 consisted of Drd2-Cre male and female mice which were counterbalanced into two groups that were injected with two different viruses in the PVT: AAV2/1-hSyn-DIO-D2R(L)-IRES-mVenus (20) and AAV2/5-hSyn-DIO-EGFP (University of North Carolina). The EGFP-expressing littermates were used as controls. Behavioral assays or histologic analysis began four weeks following viral injections. No interaction was found between sex and virus and we therefore present combined data for males and females.

### In situ hybridization

Methods were adapted from (Kellendonk et al., 2006). Brains from three-month-old C57Bl/6 mice were rapidly removed and frozen in Tissue-Tek O.C.T. mounting medium immediately following cervical dislocation. Twenty-micrometer sections were sliced using a cryostat and sections were mounted, dried at room temperature for 30 min, placed in ice-cold PFA (4%) for 5 min, rinsed with PBS for 5 min, dehydrated in 70% ethanol for 5 min, and stored in 100% ethanol at 4°C. A 45-base antisense oligonucleotide (5’ AGG CAG GGA GGC GGC AAG CAG CTG CTG TGC AGG CAA GGG GCA GAC 3’) designed to bind to the mRNA of exon 2 within the D2R was radiolabeled using a recombinant terminal transferase kit (LaRoche) and [alpha^33^P]dATP (PerkinElmer). Hybridization occurred at 42°C in a buffer containing 50% formamide, 4x SSC, and 10% dextran sulfate dissolved in DEPC-treated water. Following hybridization, slides were rinsed briefly in 1× SSC, then for 30 min in 1× SSC at 60°C, and briefly again in 1× SSC followed by 0. 1× SSC. Next, slides were dehydrated with 70% ethanol followed by 100% ethanol and allowed to dry for 30 min at room temperature before exposing to film for four weeks.

### IHC

Mice were deeply anesthetized with a mixture of ketamine (100 mg/kg) and xylazine (10 mg/kg) and perfused with PBS followed by 4% PFA. Brains were post fixed in 4% PFA for 16-24 h at 4°C. All sections were cut on a vibratome at a thickness of 50 µm and maintained at 4°C in PBS before staining. Staining followed a standard IHC protocol. Slices were incubated in a blocking buffer (0.5% BSA, 10% horse serum, 0.1% Triton X-100), washed in 0.1% Triton X-100, and incubated overnight at 4°C with the following primary antibodies as specified per experiment: chicken anti-GFP (1:1000, Abcam catalog number ab13970, RRID:AB_300798), rabbit anti-dsred (1:500, ClonTech Laboratories, catalog number 632496, RRID:AB_10013483), mouse anti-NeuN (1:200, EMD Millipore catalog number MAB377, RRID:AB_2298772), mouse anti-GAD67 (1:500, EMD Millipore catalog number MAB5406, RRID:AB_2278725), mouse anti-TH (1:750, Immunostar catalog number 22941, RRID:AB_572268), and rat anti-DAT (1:500, EMD Millipore catalog number MAB369, RRID:AB_2190413). The following secondary antibodies were used: goat anti-chicken (1:500, Thermo Fisher Scientific catalog number A11039, RRID:AB_2534096), donkey anti-rabbit (1:500, Thermo Fisher Scientific catalog number A10042, RRID:AB_2534017), donkey anti-mouse (1:500 Thermo Fisher Scientific catalog number A10036, RRID:AB_2534012), and goat anti-rat (1:500, Thermo Fisher Scientific catalog number A11006, RRID:AB_2534074). Slices were mounted with vectashield mounting media with DAPI (Vector Laboratories catalog number H-1500, RRID:AB_2336788).

For quantification of NeuN-positive neurons that coexpressed GFP in Drd2-GFP mice, 50-µm slices were sampled with every 4th section within the PVT from bregma −0.82 mm through bregma −1.70 mm according to the Paxinos and Franklin (2001) Mouse Brain Atlas.

### Imaging and image analysis

All images were acquired with either a Hamamatsu camera attached to a Carl Zeiss epifluorescence microscope or with an inverted confocal microscope (Leica LSM 700). Images were processed with NIH ImageJ software (RRID:SCR_003070) or in Adobe Photoshop.

### In vitro electrophysiology

Male and female Drd2-EGFP mice (5–14 weeks old) were used for this experiment. All *in vitro* electrophysiology was conducted in the morning hours (slicing at 8:30 A.M.). This timing was kept consistent to control for the effect of the well-established diurnal changes in PVT neurons (Kolaj et al., 2014). Mice were killed in the presence of sevoflurane and brains quickly removed and placed in ice-cold oxygenated ACSF consisting of 126 mM NaCl, 2.5 mM KCl, 2 mM MgCl2, 1.25 mM NaH_2_PO_4_, 2 mM CaCl2, 26.2 mM NaHCO3, and 10 mM D-glucose, pH 7.45, 300–310 mOsm. Several 300-µm coronal slices spanning the rostral-caudal axis of the PVT were made in ice-cold oxygenated ACSF using a vibratome. Subsequently, slices were immediately transferred to oxygenated ACSF at 32°C for 30 min followed by 30 min at room temperature. Electrodes were pulled from 1.5 mm borosilicate glass pipettes for a typical resistance of 3-6 MΩ when filled with internal solution consisting of 130 mM K-gluconate, 5 mM NaCl, 10 mM HEPES, 0.5 mM EGTA, 2 mM MgATP, 0.3 mM NaGTP, pH 7.3, 280 mOsm. The following equipment and software were used for whole-cell patch-clamp recordings: a Multiclamp 700B amplifier, a Digidata 1440A acquisition system, Clampex 10, and pClamp 10 (all from Molecular Devices). Drugs were mixed with ACSF at the following concentrations: 1 µM quinpirole hydrochloride, and 10 µM sulpiride, which are doses routinely used by us and others in slice physiology experiments.

PVT D2 neuronal recordings were conducted at room temperature using fluorescent cells (D2R-expressing) at approximately bregma −1.22 mm. Whole-cell patch-clamp recordings were performed in current-clamp mode to determine the effect of dopaminergic agonists and antagonists on cell firing. After breaking into the cell, basic cell properties were assessed in voltage clamp mode at a holding potential of -55 mV. Neurons that showed spontaneous firing were analyzed for response to D2R activation. To this end, we used gap-free current-clamp mode and added the D2R agonist quinpirole (1 µM) after 5 min of recording. Subsequently, we added the D2R antagonist sulpiride (10 µM) 8 min after initial bath application of quinpirole (13 min into the recording) to measure whether the effects of quinpirole were reversible by sulpiride. The recording was terminated 8 min following sulpiride bath application.

### Surgical procedures

Adult male and female Drd2-Cre mice were anesthetized with ketamine (100 mg/kg) and xylazine (10 mg/kg) in all surgeries except for the pseudotyped rabies tracing injections, where mice were anesthetized with 3% isoflurane. Body temperature was maintained at 37°C with a heating pad. For viral injections within the PVT, the following coordinates were used: AP (anteroposterior) = −1.1 mm, ML (mediolateral) = 0 mm, DV (dorsoventral) = −3.2 mm from bregma, which targeted middle to posterior PVT. The posterior PVT has been most extensively studied in the context of drug addiction.

We used a Nanoject II Automatic Injector (Drummond Scientific, catalog number 3-000-204) attached to a glass pipette (15-20 µm in diameter) for viral injections (1 injection per animal; total volume of 300 nl using 13 pulses of 23 nl over a 6-min injection period). We slowly retracted the pipette 5 min after completion of the injection. For the double injection of a Cre-dependent virus (AAV5-DIO-mCherry) combined with a virus that is switched off in Cre cells (AAV1-hsyn-FAS-GCaMP6f), the same total volume of virus was injected in the PVT but this volume consisted of a 1:1 mix of the two viruses.

For overexpression of D2R in the PVT, male and female Drd2-Cre mice were counterbalanced into two groups, one which received AAV2/1-hSyn-DIO-D2R(L)-IRES-mVenus (Gallo et al., 2015), and another which received AAV2/5-hSyn-DIO-EGFP (University of North Carolina) in the PVT (AP = −1.1 mm, ML = 0 mm, DV = −3.2 mm from bregma). Littermates were always used as controls. Behavioral assays or histologic analysis began four weeks following injections.

### Pseudotyped rabies single synapse retrograde tracing experiments

Adult male and female Drd2-Cre mice were used for single synapse retrograde tracing experiments. A total of 200 nl of a 2:1 mix of helper viruses rAAV5-CAG-Flex-RAB[G] (Addgene #48333) and rAAV5-EF1a-Flex-TVA-mCherry (Addgene #38044) was injected into the PVT (AP = −1.1 mm, ML = 0 mm, DV = −3.65 mm from bregma). Twelve days later, 500 nl of the pseudotyped rabies SAD-B19ΔG-mCherry (Salk viral core, EnvA G-Deleted Rabies-mCherry, Addgene #32636) was injected at the same coordinates. Mice were killed 10 d after the second injection.

### Cocaine locomotor sensitization

Mice were placed in OF boxes as described in the OF paradigm but lighting was maintained at 300-365 lux. After 90 min, mice were briefly removed from the boxes and injected intraperitoneally according to the following schedule: 2 d of saline followed by 5 d of 15 mg/kg cocaine or saline. This schedule was followed 6 d later with 2 d of injections: saline (day 13) then cocaine 15 mg/kg (day 14). Immediately following each injection, mice were returned to the OF boxes for 90 min. Mice were counterbalanced across four experimental groups according to the virus injected in the PVT as well as whether the mice received cocaine or saline across the five sensitization days: cocaine GFP_PVT_, cocaine D2R-OE_PVT_, saline GFP_PVT_, and saline D2R-OE_PVT_ (whereby OE denotes overexpression). These groups were further counterbalanced into two sub-groups, one which was run in the morning and one which was run in the early afternoon.

### Fear discrimination and contextual fear conditioning

Standard fear conditioning boxes were used in all fear conditioning experiments (Med-Associates). Freezing was recorded by overhead videos and subsequently scored using automated software (Actimetrics). Freezing bouts were included if the duration was at least 1.5 s. Automated scoring was conducted by adjusting thresholds per mouse to match each freezing bout in the video and this was done by an experimenter blind to the experimental conditions. The fear discrimination protocol was modified from (De Bundel et al., 2016).

### Day 1 (habituation)

Mice were placed in context B (plastic floor insert, plastic round wall inserts, cleaned with alcohol-based cleaning wipes) for a 2-min habituation period followed by alternating presentations of two types of tones (2.5 kHz, 7.5 kHz, 85 dB, 30 s) separated by an intertrial interval (ITI) of 20–120 s (average 66 s). A total of 10 tones were presented (5 of 2.5 kHz, 5 of 7.5 kHz).

### Day 2 (discriminative fear conditioning)

Mice were placed in context A (fear conditioning box without inserts cleaned with Virkon-S 1%) for a habituation period of 2 min followed by alternating presentations of the two tones. The mice were counterbalanced into two groups whereby one of the two types of tones [conditioned stimuli (CS)+] coterminated with a shock (unconditioned stimulus, 2 s, 0.6 mA). Mice were exposed to five CS+ and five CS- tones separated by an ITI of 20–120 s (average 66 s). Day 2 of fear conditioning occurred at 10 A.M.

### Day 3 (fear discrimination test)

The fear discrimination test day occurred in two parts. At 10 A.M., mice were placed in context B and received a habituation period of 1 min followed by four presentations of one type of tone (2.5 kHz, 85 dB, 30 s) with an ITI of 20–120 s. Four hours later, at 2 P.M., mice were returned to context B and received a habituation period of 1 min followed by four presentations of the other tone (7.5 kHz, 85 dB, 30 s) with an ITI of 20–120 s. With this behavioral design, half of the mice were exposed to the CS+ in the morning and half were exposed to the CS+ in the afternoon.

### Day 4 (contextual fear test)

Mice were returned to context A for 3 min at 10 A.M. Freezing to the context was measured during these 3 min.

### EPM

The EPM was constructed from white opaque acrylic sheets. Mice were placed in the center of the EPM facing one closed arm and allowed to explore for 5 min. Lighting was adjusted to 550-615 lux in the open arms and 350-400 lux in the closed arms and this test was conducted in the morning hours. AnyMaze software was used to track the center of each mouse and zones were drawn within this software to calculate dependent measures such as the time spent in each zone.

### OF test

Mice were placed in the corner of an open arena consisting of clear acrylic activity chambers (42 cm W × 42 cm D × 38 cm H) and allowed to explore the arena for 1 h. Lighting was maintained at 615-675 lux at the center of the OF and activity was recorded via infrared photobeams (Kinder Scientific).

### LD test

The LD test was conducted in dark rooms with single lamps above each LD apparatus. Mice were placed in the same arena as the OF test with the same software used to analyze independent measures. In addition, a dark enclosed acrylic insert was used to maintain half of the arena in darkness. Lighting for the light half was maintained at 600-650 lux and activity was recorded for 10 min via infrared bream breaks.

### PPI

Mice were placed into startle chambers and were habituated to the chambers for 5 min before any stimuli were presented. Mice were then exposed to seven types of trials: 115-dB burst of noise without a prepulse, 115 dB with a prepulse of either 2, 4, 8, 12, or 16 dB, and no noise. The program began and ended with a block of 10 trials of 115-dB pulses without a prepulse. In between these blocks of pulses were randomly interspersed presentations of the other six types of trials. The total number of trials was 100. The recording time window was 250 ms, and background noise was 70 dB.

### Operant-based paradigms

All operant-based tasks were conducted in modular test chambers (Med Associates, ENV-307W) placed within sound attenuating boxes (Med Associates, ENV-022MD).

### Outcome-specific PIT

The PIT task was performed as in Parnaudeau et al. (2015) except that sucrose pellets were used instead of grain-based pellets and 3 d of PIT testing were conducted instead of 2. In summary, mice underwent 2 d of dipper and feeder training followed by 7 d of Pavlovian training and subsequently 11 d of instrumental training. After instrumental training, mice received 3 d of PIT testing. Each of these training and testing periods is described below. These tests were followed by a PR task as well as an outcome-specific devaluation task.

### Dipper and feeder training

Mice underwent 2 d of training in which they learned to retrieve two different rewards (sucrose pellets and 20% sucrose solution) from the food magazine. This training consisted of twice daily sessions whereby one type of reward was administered during a session. For sucrose pellets, rewards were delivered on a random time schedule (average 30 s) and the sessions lasted 30 min or until 20 pellets were administered, whichever occurred first. For sucrose solution, on day 1 the dipper was raised with a drop of sucrose solution and did not retract until 10 s after the first head entry into the food magazine. These trials were separated by a variable ITI and the entire session lasted 30 min or after 20 presentations of the dipper, whichever occurred first. On day 2, the dipper was presented for 8 s regardless of a head entry and the session ended after 20 presentations of the dipper.

### Pavlovian training

Mice underwent 7 d of training whereby two CS (tone or white noise) were paired with the two food rewards (20% sucrose or sucrose pellets). During each daily 1-hour session, each 2-min CS was presented four times in a pseudorandomized fashion with a variable ITI. During each CS presentation, the food reward was delivered on a random time schedule. Mice were counterbalanced at this stage of training such that half of the mice received one pairing (i.e., tone with sucrose pellets) and the other half received the other pairing (i.e., white noise with 20% sucrose solution).

### Instrumental training

Mice underwent 11 d of training whereby one lever (i.e., left lever) was paired with one of the food rewards (i.e., sucrose pellets) while the other lever was paired with the other food reward. At this stage, mice were counterbalanced between the possible pairings. For the 11 d of training, mice received twice daily sessions to associate each of the two levers with the two food rewards. The order of the sessions was reversed daily such that a particular lever or outcome was never associated with a particular time of the day. For every session, 20 rewards or 30 min signaled the end depending on whichever occurred first. The schedule of reinforcement for the 11 d consisted of 2 d of continuous reinforcement (CRF), 3 d of random ratio (RR)5 (probability of lever press leading to a reward = 1/5), 3 d of RR10, and 3 d of RR20.

### PIT testing

PIT was measured across three consecutive days. Each session consisted of an 8-min extinction period whereby both levers were presented and no rewards or CSs were delivered. This was followed by 40 min of four presentations of each CS (2 min) separated by a 3-min fixed ITI without delivery of any food rewards.

### PR

Following PIT testing, mice were retrained on a RR20 schedule but with once daily sessions and with evaporated milk as the food reward. After 3 d of retraining, mice were tested for 2 d on a PR task following previously described methods (Carvalho Poyraz et al., 2016). This task was conducted with a schedule of reinforcement whereby the number of presses required to earn a reward doubled with each reward earned starting with a requirement of 2 lever presses for the first reward. Sessions ended after either 3 min without a lever press or after 2 h, depending on whichever occurred first.

### Outcome-specific devaluation

Following PR testing, mice were retrained with an RR20 schedule with twice daily sessions for 2 d. In one session they received sucrose pellets and in the other session a 20% sucrose solution as rewards. The same counterbalanced groups were maintained as in the PIT experiment. For example, mice for which the left lever was rewarded with sucrose pellets and the right lever was rewarded with sucrose solution during the PIT experiment were retrained with those same contingencies during RR20.

After retraining, mice underwent outcome-specific devaluation testing. Devaluation was achieved by prefeeding the mice with one of the two rewards. For instance, sucrose pellets were devalued by allowing ad libitum access to sucrose pellets for 1 h before the test. This reward was counterbalanced across the groups such that half of the mice in each lever-outcome group received one of the food rewards and the other half received the other food reward. During the actual test, lever press responses were simultaneously measured on both levers, the lever paired with the devalued reward and the lever paired with the non-devalued reward. To this end, mice were tested for 10 min in an extinction test whereby both levers were presented and no rewards were delivered.

The following day consisted of 1 day of the twice daily RR20 schedule to bring lever pressing rates back to baseline following the devaluation test under extinction conditions. Subsequently, mice underwent the second day of devaluation testing which was the same as the previous day except that the prefeeding reward was reversed.

### Drugs

Cocaine hydrochloride (Sigma, catalog number C5776) was freshly dissolved in sterile saline (1.5 mg/ml) and injected intraperitoneally at 15 mg/kg. Vehicle injections consisted of sterile saline.

### Experimental design and statistical analysis

Data were analyzed with MATLAB (The MathWorks, RRID:SCR_001622), Prism 5 (GraphPad), and StatView. Statistical tests are indicated in the results section or Table 2 and included unpaired *t* tests and repeated measures (RM) ANOVA. We used *post hoc* Bonferroni correction for follow-up individual comparisons and to account for multiple comparisons. The %PPI was calculated as the [(startle to pulse alone - startle to pulse with preceding prepulse)/startle to pulse alone] * 100%. The Pavlovian elevation score was calculated as (head entry rate during CS+) − (head entry rate during pre-CS+). The PIT transfer score ([lever press rate during the CS+] − [lever press rate during the ITIs]) was measured for both “same” (press rate measured for the lever paired with the same outcome as the CS+) and “different” (press rate measured for the lever paired with the different outcome as the CS+) levers and was averaged across the three PIT testing days. The difference between the PIT score for the same and different levers was calculated. For PR testing, breakpoint was calculated as the corresponding number of presses required for the highest ratio achieved within that session. Mice that did not press for 3 min dropped out of the experiment. For the fear conditioning tasks, freezing bouts that were equal to 1.5 s or longer were measured.

### Electrophysiology

All data were analyzed with pClamp 10 (Molecular Devices, RRID:SCR_011323). Spike frequency was calculated as the number of spikes within a 1-min period during three time points (prequinpirole: 4-5 min; postquinpirole: 10-11 min; postsulpiride: 18-19 min) whereby quinpirole was administered at 5 min and sulpiride was administered at 13 min into the recording. Membrane potential was measured just before bath application of quinpirole (5 min) and again 5.5 min after bath application of each drug (at 11.5 and 18.5 min). For both spike frequency and membrane potential, RM ANOVAs were used. We used *post hoc* Bonferroni correction for follow-up individual comparisons and to account for multiple comparisons.

## Results

### D2R-expressing neurons are concentrated along the midline of the thalamus and do not express GAD67

**Figure 1.**
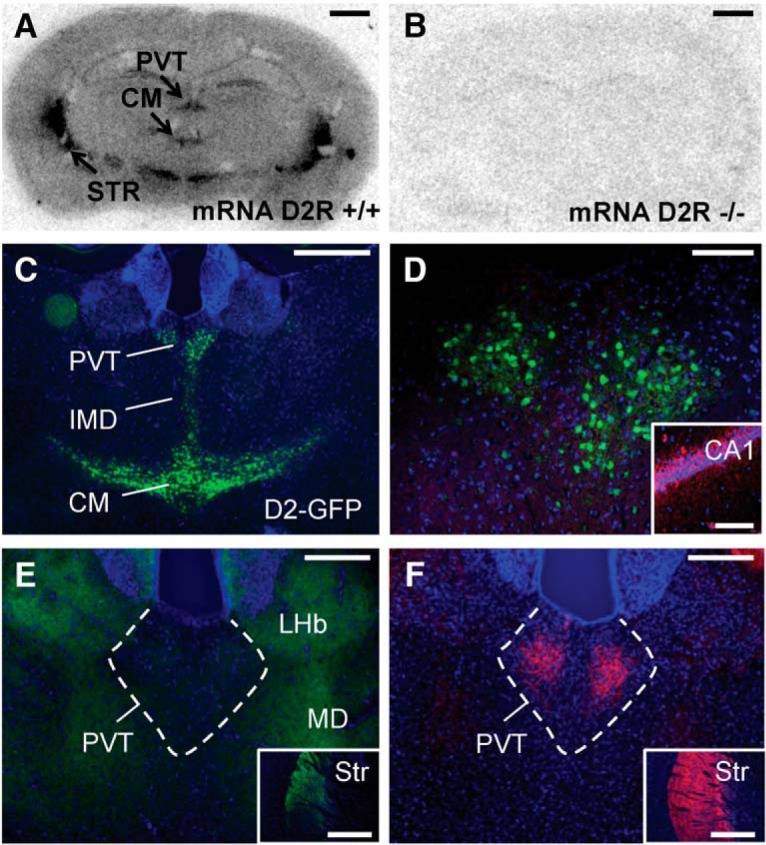
D2R-expressing neurons are concentrated in the midline thalamus where D2R expression overlaps with TH innervation in the PVT. *In situ* hybridization for D2R mRNA shows expression in the PVT and CM thalamus of wild type mice (bregma −1.7 mm; ***A***) but not in D2 knock-out mice (***B***). Scale bar: 1 mm. ***C***, IHC for GFP (green) in a Drd2-EGFP mouse shows GFP expression in the PVT, IMD, and CM thalamus. Scale bar: 500 µm. ***D***, IHC for GFP (green) and GAD67 (red) in a Drd2-EGFP mouse. No interneurons were found in the PVT. Scale bar: 100 µm. Inset, GAD67 labeling within CA1 of the hippocampus. Scale bar: 100 µm. ***E***, IHC for DAT (green) shows labeling in the MD and lateral habenula (LHb). Scale bar: 250 µm. Inset, DAT (green) labeling in the striatum. Scale bar: 1 mm. ***F***, IHC for TH (red) shows dense innervation of the PVT. Scale bar: 250 µm. Inset, TH (red) labeling in the striatum. Scale bar: 1 mm. DAPI labeling is shown in blue in ***C–F***. PVT, paraventricular nucleus of the thalamus; CM, central medial nucleus of the thalamus; STR, striatum; IMD, intermediodorsal nucleus of the thalamus; LHb, lateral habenula; MD, mediodorsal nucleus of the thalamus.

In humans and postnatal mice, thalamic D2Rs are most densely expressed in the midline thalamus (Hurd et al., 2001; Rieck et al., 2004; Yuge et al., 2011). In a first step, we determined whether enriched midline expression of D2R is also observed in the adult mouse. *In situ* hybridization for D2R mRNA in wild type mice densely labeled the PVT in middle to posterior PVT sections (bregma −0.94 mm through −2.18 mm) as well as in the central medial (CM) nucleus. No labeling was observed in D2R knock-out mice (Fig. 1*A*,*B*). Using a D3R-specific probe we also observed some limited D3R expression in the PVT (data not shown).

We used Drd2-EGFP mice to obtain an independent measure of *Drd2* gene transcriptional activity. Several medial, midline, and intralaminar thalamic nuclei showed GFP expression in Drd2-EGFP mice. These included the PVT, intermediodorsal (IMD), CM, paracentral (PC), interanteromedial (IAM), anteromedial (AM), and posteromedian (PoMn) thalamic nuclei. There was also scattered GFP expression in the centrolateral (CL) and mediodorsal (MD) thalamic nuclei, with stronger expression notable in the medial MD compared to the central and lateral MD. Within the PVT, D2R-expressing cells were absent in the most anterior portions of the anterior PVT (aPVT) with more dense expression in the middle and posterior PVT. A representative coronal slice including the PVT, IMD, and CM is shown in Fig. 1*C*.

We then performed IHC for GAD67 in Drd2-EGFP mice and found that the midline thalamus is devoid of interneurons (Fig. 1*D*).

### Fibers immunoreactive for TH but not DAT innervate the PVT

Since different midline thalamic nuclei have different functions (Vertes et al., 2015), we focused our subsequent analysis on one of these nuclei, the PVT. We first determined whether the PVT receives DA innervation. IHC for the DA transporter (DAT) that is expressed in a subpopulation of dopaminergic neurons of the ventral tegmental area (VTA), substantia nigra (SN), and hypothalamus revealed a lack of innervation by DAT+ fibers (Fig. 1*E*). In marked contrast, IHC for tyrosine hydroxylase (TH), which labels dopaminergic as well as noradrenergic neurons, revealed strong innervation of the PVT by TH+ fibers (Fig. 1*F*).

### A D2R agonist inhibits tonic firing in D2R-expressing PVT neurons while a D2R antagonist reverses this inhibition

**Figure 2.**
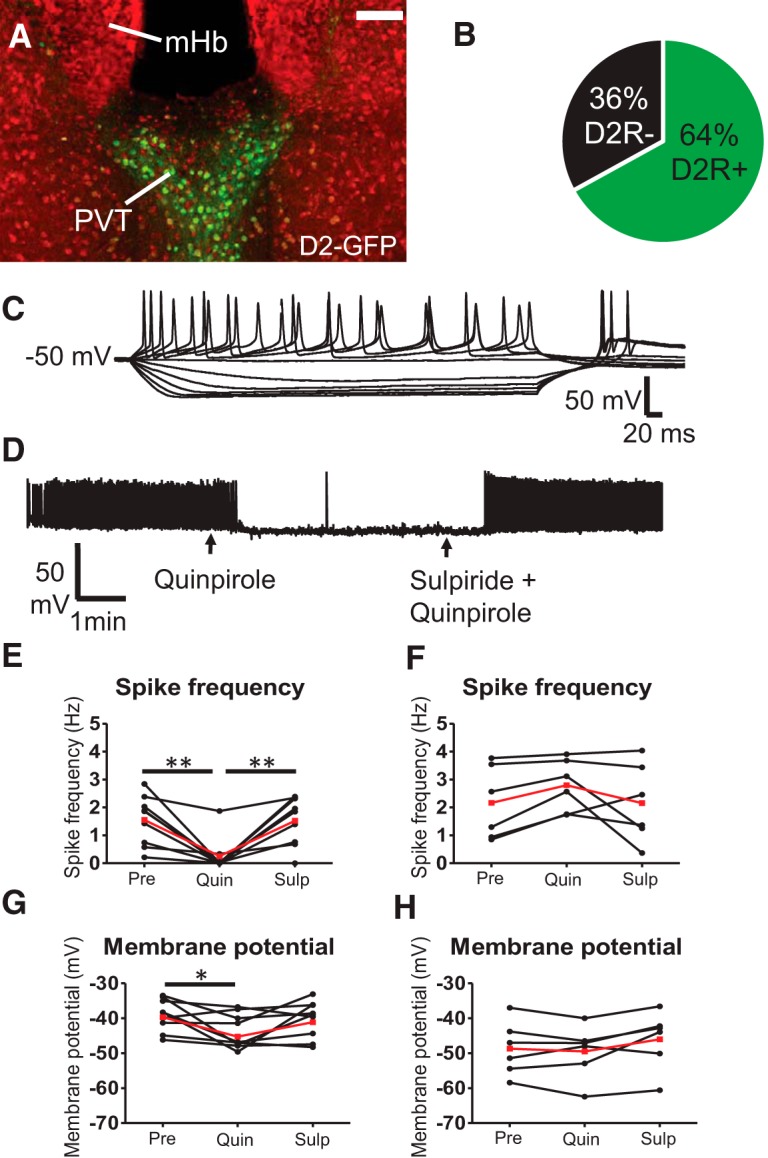
D2R activation in D2R-expressing PVT neurons induces an inhibition of tonic firing which is reversed by D2R antagonism. ***A***, IHC for NeuN (red) and GFP (green) in the PVT in a Drd2-EGFP mouse. Scale bar: 100 µm. ***B***, A total of 64% of PVT neurons express GFP in Drd2-EGFP mice. ***C***, All GFP-expressing PVT neurons exhibited a low-threshold rebound spike following recovery to baseline membrane potential from hyperpolarizing injections of current. ***D***, Example trace of a tonically active D2R-expressing PVT neuron. Quinpirole induced an inhibition of action potential firing that was reversed by coapplication of sulpiride. ***E***, Quantification of firing frequency GFP+ neurons. RM ANOVA: *F*_drug(2,8)_ = 12.04, *p* = 0.0006, **p < 0.01 Bonferroni *post hoc*. ***F***, Quantification of firing frequency GFP− neurons. RM ANOVA: *F*_drug(2,6)_ = 2.65, *p* = 0.12. ***G***, Quantification of resting membrane potential GFP+ neurons. RM ANOVA *F*_drug(2,8)_ = 4.0, *p* = 0.039, *p < 0.05 Bonferroni *post hoc*. ***H***, Quantification of resting membrane potential GFP− neurons. RM ANOVA *F*_drug(2,6)_ = 3.94, *p* = 0.048. Black data points show individual mice and red data points show the mean in ***E–H***. PVT, paraventricular nucleus of the thalamus; mHb, medial habenula.

We quantified the percentage of PVT neurons that express D2R by performing dual IHC for NeuN and GFP in Drd2-EGFP mice within bregma −0.82 mm through bregma −1.70 mm (Paxinos and Franklin, 2001) and found that 64% of neurons expressed GFP (Fig. 2*A*,*B*).

Next, we performed whole-cell patch-clamp recordings from fluorescently labeled cells in the PVT of Drd2-EGFP mice (near bregma -1.22 mm). All GFP+ neurons showed the induction of a low-threshold rebound spike following hyperpolarizing injections of current, which is a characteristic of thalamic relay neurons (Fig. 2*C*; Rhodes and Llinás, 2005). Considerable heterogeneity of PVT neuronal firing patterns has been described previously whereby multiple types of activity patterns were observed in aPVT neurons following current injection (Yeoh et al., 2014). In this study, the majority of aPVT neurons showed tonic firing (47%) or burst firing (21%) and an additional smaller percentage of neurons exhibited single spiking (18%), delayed firing (11%), and reluctant firing (3%; Yeoh et al., 2014). We similarly noticed considerable heterogeneity in the firing patterns of GFP+ PVT neurons and therefore decided to focus our study on determining the effect of D2R activation on tonically active D2R-expressing PVT neurons. We found that 48% of GFP+ PVT neurons were tonically active in current-clamp mode (11 of 23). Bath application of the D2R agonist quinpirole (1 µM) decreased spike frequency in this neuronal population by 83%. This effect was reversed after subsequent coapplication of the D2R antagonist sulpiride (10 µM, RM ANOVA: *F*_drug(2,8)_ = 12.04, *p* = 0.0006, *n* = 9, Bonferroni *post hoc*: baseline vs quinpirole *p* < 0.01, quinpirole vs sulpiride *p* < 0.01; Fig. 2*D*, 2*E*). We also observed that quinpirole hyperpolarized the resting membrane potential by 4.8 mV, which was reversed by subsequent coapplication of sulpiride (RM ANOVA: *F*_drug(2,8)_ = 4.0, *p* = 0.0392, Bonferroni *post hoc*: baseline vs quinpirole *p* < 0.05; Fig. 2*G*).

To determine if quinpirole affects firing rates in D2R-negative neurons we also recorded from GFP- neurons. We found that 30% of GFP- PVT neurons were tonically active in current-clamp mode (6 of 20). No changes in firing rates were observed in these neurons (RM ANOVA: *F*_drug(2,6)_ = 2.65, *p* = 012, *n* = 6, with a minimal effect on resting membrane potential (-0.8 mV; RM ANOVA: *F*_drug(2,6)_ = 3.94, *p* = 0.048, *n* = 6, Bonferroni *post hoc* comparisons *p* > 0.05; Fig. 2*F*, *H*).

### D2R-expressing PVT neurons innervate regions of the limbic system

**Figure 3.**
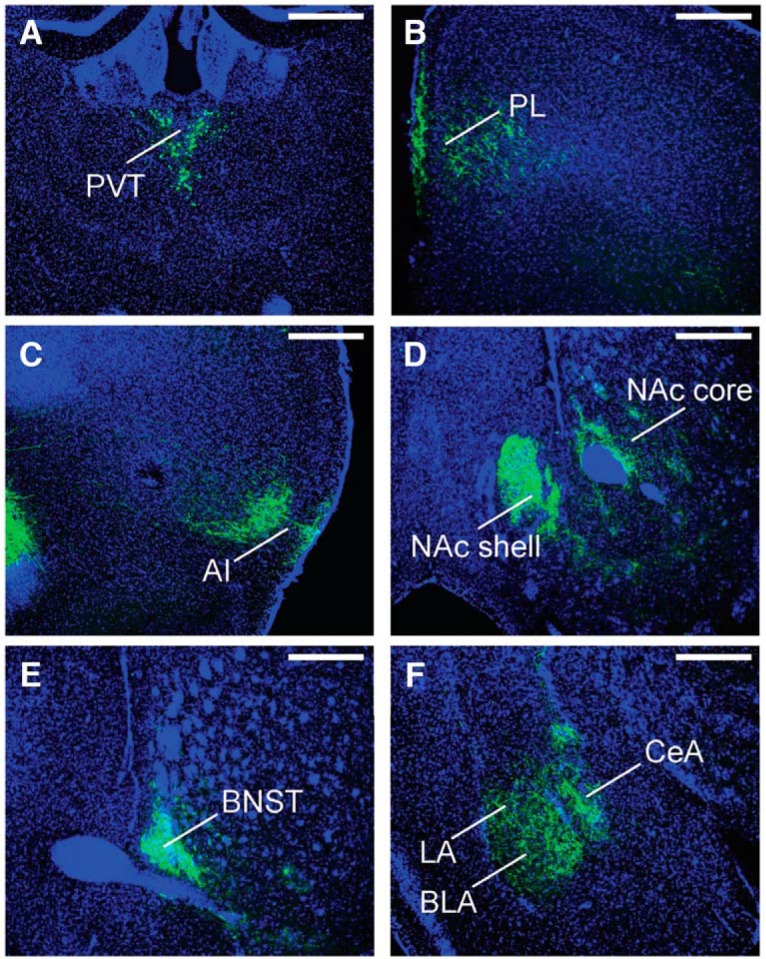
D2R-expressing PVT neurons send projections to multiple regions within the limbic system. ***A***, Site of injection of AAV5-DIO-eYFP virus in the PVT of a Drd2-Cre mouse with few D2R-positive neurons in the medial MD and IMD. D2R-expressing PVT neurons project strongly to the PL (***B***), AI (***C***), NAc (***D***), BNST (***E***), lateral amygdala (LA), basolateral amygdala (BLA), and CeA (***F***). Scale bars: 500 µm. PVT, paraventricular nucleus of the thalamus; PL, prelimbic cortex; Al, agranular insular cortex; NAc, nucleus accumbens; BNST, bed nucleus of the stria terminalisl; CeA, central nucleus of the amygdala.

D2R-expressing PVT neuronal efferents were identified with injections of Cre-dependent AAV5-DIO-eYFP in the PVT of Drd2-Cre mice (Fig. 3*A*). Fibers immunoreactive for GFP were identified brainwide in three mice and target regions are listed in Table 1. Strong innervation was found in the prelimbic (PL; Fig. 3*B*), agranular insular (AI; Fig. 3*C*) cortices, NAc (Fig. 3*D*), bed nucleus of the stria terminalis (BNST; Fig. 3*E*), amygdala (Fig. 3*F*), and the interstitial nucleus of the posterior limb of the anterior commissure (IPAC; data not shown). Within the NAc, labeling was patchy and terminals were most dense in the medial shell (Fig. 3*D*). Within the amygdala, fibers were found in the lateral, basal, and central nuclei (Fig. 3*F*). We observed sparser innervation of the caudate putamen and entorhinal cortex.

**Table 1.** Output and input projections to D2R-expressing neurons in the PVT

	Output projections	Input projections
AI	+	+
PL	+	+
Orbital frontal cortex	+	+
Cingulate cortex	−	+
NAc (core and shell)	+	+
Caudate putamen	+	+
BNST	+	+
Lateral septum	−	+
IPAC	+	+
Amygdala	Central, lateral, basolateral	Central
Zona incerta	−	+
Reticular thalamus	−	+
Hypothalamus: preoptic, ventromedial, anterior, lateral, posterior, dorsomedial, arcuate, suprachiasmatic, paraventricular, A14, and supramammillary nuclei	−	+
PAG	−	+
VTA	−	+
SN	−	+
LC	−	+
LPBN	−	+
Raphe (dorsal and raphe magnus)	−	+

Al, agranular insular cortex; PL, prelimbic cortex; NAc, nucleus accumbens; BSNT, bed nucleus of the stria terminalis; IPAC, interstitial nucleus of the posterior limb of the anterior commissure; PAG, periaqueductal gray; VTA, ventral tegmental area; SN, substantia nigra; LC, locus coeruleus; LPBN, lateral parabrachial nucleus.

**Figure 4.**
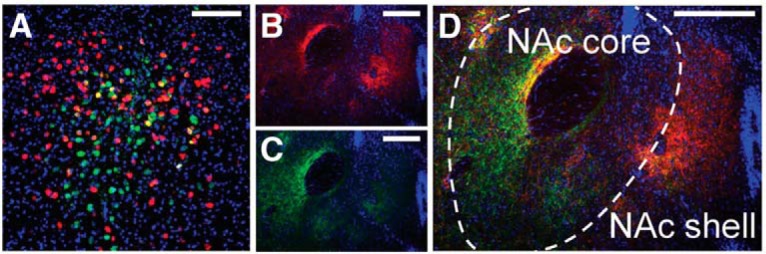
The relative density of D2R-positive and D2R-negative PVT neuronal projections differs within the NAc. ***A***, PVT site of dual injection of AAV5-DIO-mCherry (red) and AAV1-hsyn-FAS-GCaMP6f (green) in a Drd2-Cre mouse. Scale bar: 100 µm. ***B***, Cre-positive PVT neuronal projections within the NAc (red). Scale bar: 500 µm. ***C***, Cre-negative PVT neuronal projections within the NAc (green). Scale bar: 500 µm. ***D***, The overlap between Cre-positive and Cre-negative PVT neuronal projections within the NAc. Scale bar: 500 µm. NAc, nucleus accumbens.

We next investigated whether PVT D2R-negative neurons project to the same brain regions as D2R-positive neurons. To this end, we labeled D2R-positive neurons in red and D2R-negative neurons in green by injecting a combination of AAV5-DIO-mCherry with AAV1-hsyn-FAS-GCaMP6f, thereby combining a Cre-On (DIO) with a Cre-Off (FAS) strategy in the same animal. Only a small percentage of cells were colabeled (7.5%), which indicates that our mutually exclusive dual viral injection strategy was effective. The combined injection of the viruses revealed a general overlap of projection targets suggesting no qualitative differences (data not shown). However, while there was considerable overlap of the projections within the NAc, fibers labeled red by AAV5-DIO-mCherry (D2R-positive PVT projections) were more concentrated in the medial NAc shell whereas fibers labeled green by AAV1-hsyn-FAS-GCaMP6f (D2R-negative projections) were more concentrated in the NAc core (Fig. 4).

### D2R-expressing PVT neurons show reciprocal and nonreciprocal afferent projections

**Figure 5.**
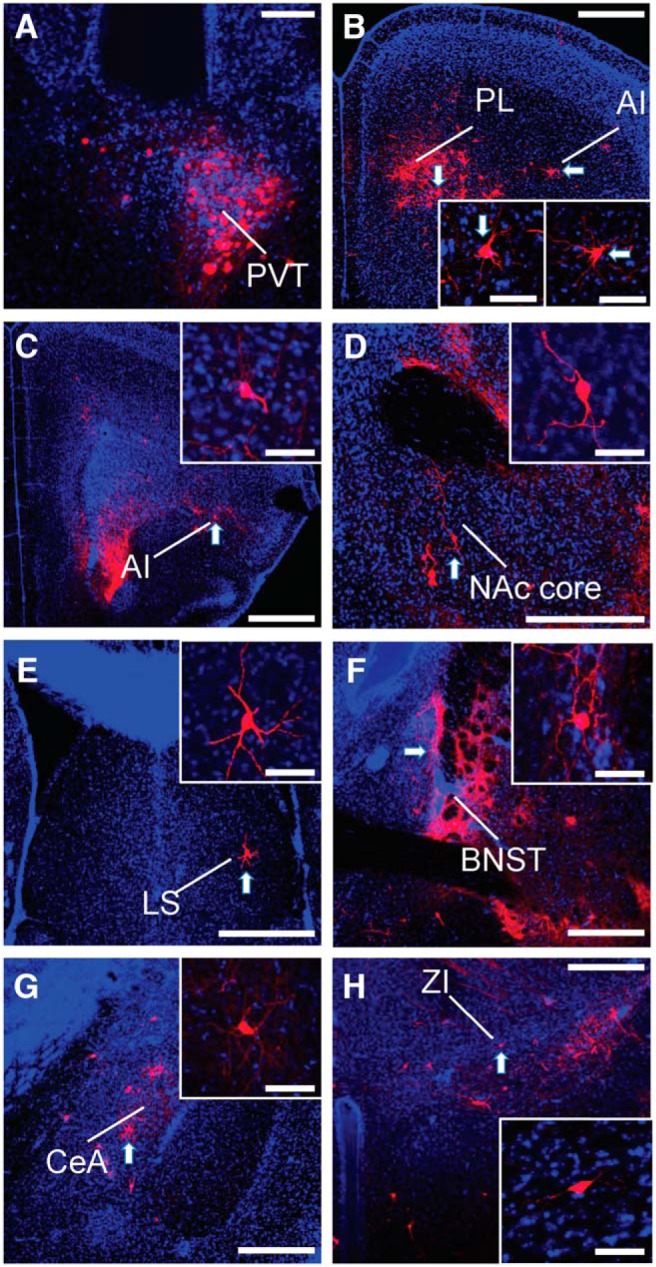
D2R-expressing PVT neurons receive inputs from many regions within the limbic system. ***A***, Site of pseudotyped rabies viral injection within the PVT of a Drd2-Cre mouse. Scale bar: 100 µm. Cell bodies were retrogradely labeled in PL and AI (***B***, ***C***), NAc core (***D***), lateral septum (LS; ***E*)**, BNST (***F***), CeA (***G***), and zona incerta (ZI; ***H***). Scale bars for ***B–H***: 500 µm. Insets show individual cell bodies retrogradely labeled by the pseudotyped rabies virus within each region. Scale bars for insets: 50 µm. PVT, paraventricular nucleus of the thalamus; PL, prelimbic cortex; Al, agranular insular cortex; NAc, nucleus accumbens; BNST, bed nucleus of the stria terminalis; CeA, central nucleus of the amygdala.

We identified incoming projections to D2R-expressing neurons of the PVT using pseudotyped rabies retrograde tracing experiments in Drd2-Cre mice. We found reciprocal and non-reciprocal innervation of the D2R-positive PVT neurons (Table 1). Regions which reciprocally innervated these neurons included AI, PL, and orbital frontal cortex, the NAc, caudate putamen, BNST, IPAC, and central amygdala. Non-reciprocal innervation arose from regions including the cingulate cortex, lateral septum, basal and lateral amygdala, zona incerta, reticular thalamus, hypothalamic regions (preoptic, ventromedial, anterior, lateral, posterior, dorsomedial, arcuate, suprachiasmatic, A14, and supramammillary nuclei), the PAG, VTA, SN, LC, lateral parabrachial nucleus (LPBN), and raphe nuclei. Projections from PL, orbital, and AI cortex, the NAc core, lateral septum, BNST, central amygdala, and zona incerta are shown in Figure 5.

In Figure 1, we have shown that the PVT receives DAT-negative, TH-positive fibers. TH-positive fibers may arise from DAT-negative dopaminergic neurons in the hypothalamus (ventro-rostral A10, A11, A13, A15 DA cell groups), midbrain (dorsocaudal A10 embedded in the PAG), or noradrenergic neurons of the rostral medulla (C1-3) and LC (Chen and Su, 1990; Phillipson and Bohn, 1994; Otake and Ruggiero, 1995; Krout et al., 2002; Li et al., 2014a). Although we found retrogradely labeled neurons in the VTA, PAG, and hypothalamus, none were TH-positive (data not shown).

Based on the identified neural circuitry, we designed a battery of behavioral tests to determine the role of PVT D2Rs in behaviors that have been shown to involve either the PVT or two main interconnected structures, the amygdala and NAc.

### Overexpression of D2R in D2R-expressing PVT neurons attenuates cocaine locomotor sensitization

**Figure 6.**
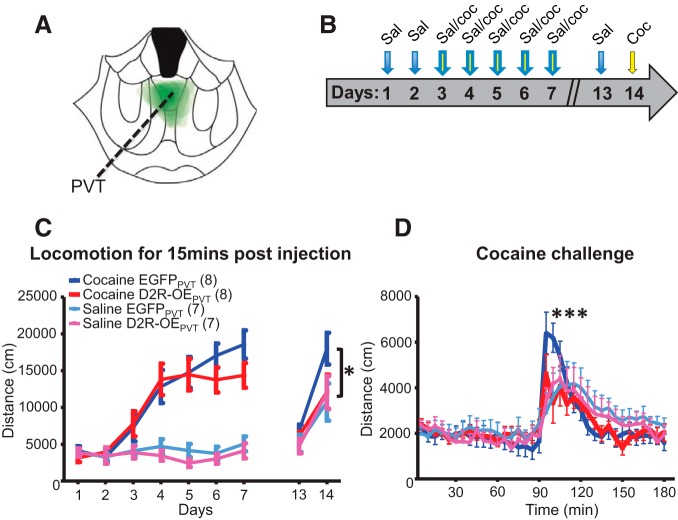
Overexpression of D2R in D2R-expressing PVT neurons inhibits cocaine locomotor sensitization. ***A***, Viral spread of AAV2/1-hSyn-DIO-D2R(L)-IRES-mVenus in all D2R-OE_PVT_ mice within the cocaine behavioral experiment overlaid on a single coronal image (1.2 mm posterior to bregma). ***B***, Illustration of the cocaine locomotor sensitization behavioral design. ***C***, D2R-OE_PVT_ mice receiving cocaine injections exhibited decreased locomotion for the first 15 min on day 14, the cocaine challenge day, when compared to control EGFP_PVT_ mice receiving cocaine injections (RM ANOVA over days 1–14: *F*_groupxday(3,24)_ = 8.6, *p* < 0.0001; *post hoc* Bonferroni D2R-OE_PVT_ vs EGFP_PVT_ on day 14 *p < 0.05, *n* = 7-8/group). ***D***, D2R-OE_PVT_ mice receiving cocaine injections exhibited decreased locomotion immediately after the injection when analyzed over the entire 180-min period (RM ANOVA: *F*_groupxtime(3105)_ = 1.82, *p* < 0.0001, Bonferroni *post hoc*: ***p < 0.001 for 20th 5-min time bin for D2R-OE_PVT_ vs EGFP_PVT_ with cocaine, *n* = 7-8/group). PVT, paraventricular nucleus of the thalamus.

PVT lesions impair locomotor sensitization to cocaine (Young and Deutch, 1998). Additionally, the NAc, a major target region of the PVT, has a well-established role in cocaine sensitization (Pierce and Kalivas, 1997). We therefore determined whether PVT D2Rs modulate cocaine sensitization. To this end, we upregulated D2R expression selectively in the PVT with the aim of enhancing D2R signaling in response to endogenous DA release (Fig. 6*A*). Upregulation was obtained by injecting a Cre-dependent AAV2/1 virus expressing D2R and mVenus in the PVT of Drd2-Cre mice. Using a similar approach in the NAc, we have recently shown that viral-mediated upregulation leads to a 3-fold increase in D2R membrane binding in the NAc (Gallo et al., 2015). Moreover, virally expressed D2Rs couple to G proteins in a striatal GTPγS assay (P. Donthamsetti, E.F. Gallo, C. Kellendonk, J.A. Javitch, unpublished observations) and inhibit synaptic transmission at indirect pathway output synapses (E.F. Gallo, J.A. Javitch, C. Kellendonk, unpublished observations), thereby verifying functionality of the construct *in vivo*.

In the cocaine locomotor sensitization task, mice were first habituated for 2 d with saline injections and then injected with 15 mg/kg cocaine or with the same volume of saline once per day across 5 d. On days 13 and 14, mice received saline and cocaine injections, respectively (Fig. 6*B*). We analyzed locomotor activity for 15 min following injections. Both D2R-OE_PVT_ and EGFP_PVT_ mice showed comparable locomotor activity during the 5 d of cocaine injections. However, during the cocaine challenge day (day 14), cocaine-pretreated D2R-OE_PVT_ mice showed a significantly inhibited response to cocaine (RM ANOVA over days 1–14: *F*_groupxday(3,24)_ = 8.6, *p* < 0.0001; *post hoc* Bonferroni cocaine-pretreated D2R-OE_PVT_ vs cocaine-pretreated EGFP_PVT_ on day 14 *p* < 0.05, *n* = 7-8/group; Fig. 6*C*). We followed up with an analysis over the entire course of the 180 min of the cocaine challenge day and measured a group by time interaction. *Post hoc* analysis revealed a decrease in cocaine-induced locomotion in D2R-OE_PVT_ mice directly after injections (RM ANOVA: *F*_groupxtime(3105)_ = 1.82, *p* < 0.0001, Bonferroni *post hoc*: *p* < 0.001 for 20th time bin for cocaine-pretreated D2R-OE_PVT_ vs cocaine-pretreated EGFP_PVT_, *n* = 7-8/group; Fig. 6*D*).

### Overexpression of D2Rs in D2R-expressing PVT neurons does not affect fear acquisition, discrimination, or contextual fear expression

Several publications have demonstrated a role of the PVT in fear conditioning (Li et al., 2014b; Do-Monte et al., 2015; Penzo et al., 2015) and we therefore determined whether upregulation of D2Rs affects fear conditioning.

**Figure 7.**
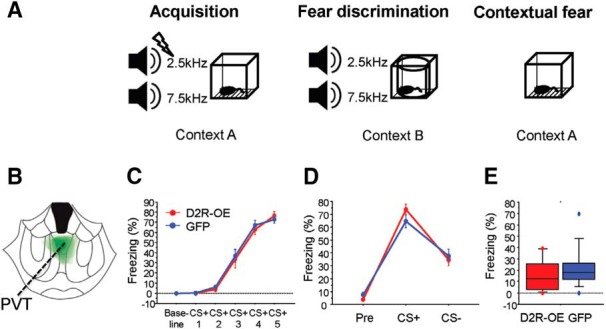
Overexpression of D2R in D2R-expressing PVT neurons does not affect fear conditioning. ***A***, Diagram illustrating the fear discrimination paradigm. ***B***, Viral spread of AAV2/1-hSyn-DIO-D2R(L)-IRES-mVenus in D2R-OE_PVT_ mice overlaid on a single coronal image (1.2 mm posterior to bregma). ***C***, D2R-OE_PVT_ and control EGFP_PVT_ mice exhibited similar levels of freezing both at baseline and to the CS+ during acquisition (RM ANOVA: *F*_virus(1,26)_ = 0.08, *p* = 0.77; *F*_virusxtime(1,5)_ = 0.25, *p* = 0.94). ***D***, D2R-OE_PVT_ and control EGFP_PVT_ mice exhibited similar levels of fear discrimination between the two tones (RM ANOVA: *F*_virus(1,26)_ = 0.04, *p* = 0.85; *F*_virusxstimulus(1,2)_ = 1.7, *p* = 0.08). ***E***, D2R-OE_PVT_ mice exhibited similar levels of freezing on reexposure to context ***A*** when compared to control EGFP_PVT_ mice (D2R-OE_PVT_ = 15.7 ± 3.7%, EGFP_PVT_ = 21.6 ± 4.7, *t* test: *p* = 0.32, *n* = 14/14). PVT, paraventricular nucleus of the thalamus.

Mice were trained with two tones (2.5 and 7.5 kHz), one predicting a shock (CS+) and one unpaired with the shock (CS-), and received five presentations of each (acquisition; Fig. 7*A*, left). On the subsequent day, cued fear discrimination was tested by presenting the two tones in a different context (Fig. 7*A*, middle). On the final day, contextual fear expression was tested by exposing the mice to the context used during acquisition (Fig. 7*A*, right). We measured no differences between D2R-OE_PVT_ mice (Fig. 7*B*) and control EGFP_PVT_ mice either in baseline levels of freezing or in freezing during the five CS+ presentations during acquisition (RM ANOVA: *F*_virus(1,26)_ = 0.08, *p* = 0.77; *F*_virusxtime(1,5)_ = 0.25, *p* = 0.94; Fig. 7*C*). During fear discrimination testing, D2R-OE_PVT_ and control mice exhibited a similar level of freezing to the new context and their freezing response was comparable both for the CS+ and CS- tones (RM ANOVA: *F*_virus(1,26)_ = 0.04, *p* = 0.85; *F*_virusxstimulus(1,2)_ = 1.7, *p* = 0.08; Fig. 7*D*). Moreover, D2R-OE_PVT_ and EGFP_PVT_ mice did not differ in the contextual fear recall test (D2R-OE_PVT_ = 15.7 ± 3.7%, EGFP_PVT_ = 21.6 ± 4.7, *t* test: *p* = 0.32, *n* = 14/14; Fig. 7*E*).

### Overexpression of D2Rs in D2R-expressing PVT neurons does not affect measures of motivation, anxiety, and sensorimotor gating

Because of the important role of the NAc in both motivated behavior (Carvalho Poyraz et al., 2016) as well as in the ability of Pavlovian stimuli to guide instrumental behavior (Corbit and Balleine, 2011), we tested D2R-OE_PVT_ mice in several operant-based tasks addressing motivated behavior. These tasks included outcome-specific PIT, a PR task for food, and instrumental responding after outcome-specific devaluation of a food reward. D2R-OE_PVT_ mice did not differ from EGFP_PVT_ mice in any of these tasks. Furthermore, D2R overexpression in the PVT did not affect measures of anxiety and locomotor activity in the OF and EPM, nor did it affect sensorimotor gating, as measured by PPI. The results of these unaffected behavioral outcome measures are summarized in Table 2.

**Table 2.** Summary of behaviors not affected by D2R upregulation in the PVT

Task	Outcome measure	D2R-OE_PVT_	EGFP_PVT_	Statistics
OF test	Ambulatory distance	207 m/60 min	215 m/60 min	RM ANOVA: *F*Virus(1,14) = 0.211, *p* = 0.65, *n* = 8/8
	Time in center	455 ± 176 s of 60 min	440 ± 238 s of 60 min	RM ANOVA: *F*Virus(1,14) = 0.02, *p* = 0.88, *n* = 8/8
EPM	Open arm time	147.0 ± 8.4 s	140.7 ± 11.1 s	*t* test: *p* = 0.65, *n* = 8/8
	Closed arm time	109.9 ± 7.3 s	110.5 ± 7.2 s	*t* test: *p* = 0.96, *n* = 8/8
LD test	Time in light zone (first 5 min)	130.3 ± 6.7 s	126.0 ± 9.4 s	*t* test: *p* = 0.71, *n* = 8/8
PPI	Startle response	0.32 ± 0.29	0.25 ± 0.09	*t* test: *p* = 0.53, *n* = 8/8
	PPI	Mean PPI (pp2,4,8,12,16): 21.3%	Mean PPI (pp2,4,8,12,16): 20.4%	RM ANOVA: *F*virus(1,14) = 0.01 *p* = 0.92; *F*virusxprepulse(4,14) = 0.51, *p* = 0.72, *n* = 8/8
PIT	Pavlovian learning: dipper approach	No effect of virus		RM ANOVA: *F*virus(1,14) = 2.6E-5, *p* = 0.99; *F*virusxday(1,6) = 0.22, *p* = 0.96
(outcome specific)	Instrumental response for sucrose solution	No effect of virus		RM ANOVA: *F*virus(1,14) = 0.52, *p* = 0.48; *F*virusxday(1,10) = 1.5, *p* = 0.14
	Instrumental response for pellets	No effect of virus		RM ANOVA: *F*virus(1,14) = 0.01, *p* = 0.91; *F*virusxday(1,10) = 0.4, *p* = 0.92
	Pavlovian transfer score	Same-different: 2.34 ± 1.588	Same-different: 0.79 ± 1.87	ANOVA: *F*virus(1,14) = 3.2, *p* = 0.10, *n* = 8/8
PR	Break point	380.0 ± 69.6 presses	488.0 ± 82.9 presses	*t* test: *p* = 0.33
	Time until drop out	77.4 ± 11.6 min	74.8 ± 9.5 min	*t* test: *p* = 0.86
Devaluation	Lever press rate: valued–devalued lever	2.00 ± 0.70 presses/min over 10 min	2.30 ± 0.84 presses/min over 10 min	RM ANOVA: *F*virus(1,14) = 0.057, *p* = 0.81; *F*virusxday(1,9) = 0.59, *p* = 0.80

OF, open field; EPM, elevated plus maze; LD, light-dark; PPI, prepulse inhibition; pp, preplus; PIT, Pavlovian-to-instrumental tranfer; PR, progressive ratio.

## Discussion

Here, we describe enriched expression of D2Rs in the midline thalamus of the adult mouse and show that activation of these receptors inhibits firing of PVT relay neurons. Using anterograde and retrograde tracing studies, we identified that D2R-expressing neurons in the PVT are heavily interconnected with areas of the limbic system including the prefrontal cortex, NAc, and amygdala. Finally, we found that selective upregulation of D2Rs in the PVT impairs cocaine locomotor sensitization.

### The midline thalamus expresses functional D2Rs

Using *in situ* hybridization and Drd2-EGFP mice, we found that D2Rs are preferentially expressed in midline thalamic nuclei, which is in agreement with observations in humans (Hurd et al., 2001; Rieck et al., 2004). Approximately two-thirds of PVT neurons express D2Rs and the D2R agonist quinpirole inhibited spontaneous firing, an effect reversed by D2R antagonism.

The mechanism by which D2R agonists inhibit tonic firing of PVT neurons remains unknown. In the mediodorsal thalamus, quinpirole has been shown to elicit mixed effects, hyperpolarizing some neurons and depolarizing others (Lavin and Grace, 1998). In the dorsal lateral geniculate nucleus, quinpirole application can inhibit or enhance relay neuron activity and GABA-A receptors were found to mediate the inhibitory effects (Albrecht et al., 1996; Zhao et al., 2002). Furthermore, quinpirole increases spontaneous inhibitory currents in relay neurons of the dorsal lateral geniculate nucleus (Munsch et al., 2005). However, since the PVT is devoid of interneurons (Fig. 1*D*), GABAergic effects may not explain the results of our study. One limitation of the previous studies is that they could not determine if recorded neurons expressed D2Rs, thereby making the distinction between direct and indirect effects more difficult.

To avoid this limitation, we recorded from genetically identified D2R-expressing neurons. Most likely, D2R activation inhibits tonic firing via Gi-protein-mediated activation of GIRK channels, as has been described for dopaminergic neurons in the midbrain (Lacey et al., 1987; McCall et al., 2017). Consistent with this, PVT neurons express functional GIRK channels (Saenz del Burgo et al., 2008; Hermes et al., 2013; Zhang et al., 2013; Kolaj et al., 2014).

### D2R-expressing PVT neurons are heavily interconnected with limbic circuits

Using anterograde tracing methods in D2R-expressing PVT neurons, we found dense projections to regions of the limbic system including PL and AI cortices, the NAc, amygdala, and BNST (Fujiyama et al., 2006; Parsons et al., 2007; Unzai et al., 2015). Within the NAc, labeling was stronger in the medial shell and sparser in the core. We then determined whether D2R-positive and D2R-negative PVT neurons project to different brain areas. Whereas both cell populations project to the same brain regions, we found differences in the relative density of projections within the NAc: D2R-positive neurons more densely targeted the medial shell while D2R-negative neurons more densely targeted the core. PVT D2Rs may therefore more readily affect behaviors that are sensitive to NAc shell function.

To determine the regions that innervate D2R-expressing PVT neurons, we injected the pseudotyped rabies strain SAD-B19ΔG (Wall et al., 2013) into the PVT of Drd2-Cre mice. In addition to providing cell specificity, this approach has the additional benefit that neighboring nuclei such as the habenula do not contaminate the tracing study. We found that numerous limbic regions innervate D2R-expressing PVT neurons (Table 1), including PL and AI cortices, the NAc, amygdala, and BNST. In addition, we identified projections from orbital, and cingulate cortices, the caudate putamen, lateral septum, IPAC, zona incerta, reticular thalamus, PAG, VTA, SN, LC, LPBN, and raphe nuclei, as well as various hypothalamic nuclei.

Our cell type-specific anterograde and retrograde tracing studies thus establish that D2R-expressing PVT neurons are reciprocally innervated by numerous regions of the limbic system but receive additional non-reciprocal inputs mostly from the hypothalamus and brainstem nuclei. This is comparable to what has been observed in rats using conventional tracers, suggesting that D2R-expressing neurons do not qualitatively differ in their connectivity from other PVT neurons (Krout et al., 2002; Li and Kirouac, 2012; Colavito et al., 2015; Vertes et al., 2015).

We additionally attempted to identify the dopaminergic innervation of D2R-expressing PVT neurons by colabeling virally infected neurons with TH. Surprisingly, we found no overlap between the pseudotyped rabies-labeled neurons and TH staining. While virally infected cells were present in dopaminergic regions, these cells did not express TH. This is despite robust levels of TH terminal expression in the PVT (Fig. 1) and previous work demonstrating innervation of the PVT by the hypothalamus, PAG, and LC of the rat (Otake and Ruggiero, 1995; Li et al., 2014a). We believe that this discrepancy is due to the low efficiency of transsynaptic rabies uptake by dopaminergic neurons as has been recently described for pseudotyped rabies virus (Wall et al., 2013).

### PVT D2Rs attenuate cocaine locomotor sensitization

After identifying the neural circuitry of D2R-expressing PVT neurons, we designed a battery of behavioral tests addressing behaviors known to be supported by both the NAc and the amygdala. We further selected tasks with relevance for drug addiction (cocaine sensitization) and the negative symptoms of schizophrenia (sensorimotor gating and behaviors assessing motivation). Due to the established roles of the PVT and amygdala in fear conditioning, we also measured fear conditioning.

Selective upregulation of D2Rs in the PVT did not affect anxiety, sensorimotor gating, or the motivation to work for food. Moreover, instrumental and Pavlovian learning, as well as PIT, were unaltered in these mice. This was despite the prominent projections of D2R-expressing PVT neurons to the NAc shell, a region which is strongly implicated in outcome-specific PIT (Corbit and Balleine, 2011). In contrast, PVT D2R overexpression inhibited cocaine locomotor sensitization.

There is accumulating evidence that the PVT is part of the circuitry involved in drug addiction. Early investigations showed that the PVT supports intracranial self-stimulation (Clavier and Gerfen, 1982) and cocaine or cues previously paired with cocaine induce c-Fos expression in the PVT (Brown et al., 1992; Deutch et al., 1998; Matzeu et al., 2015b). Lesion and inactivation studies additionally identified the importance of the PVT for locomotor sensitization to cocaine and for conditioned place preference (Young and Deutch, 1998; Browning et al., 2014). The PVT also has strong projections to the NAc shell, which promotes cocaine seeking behavior (Figs. 3, 4; Berendse and Groenewegen, 1990; Pinto et al., 2003) and disrupting synaptic transmission of projections from the PVT to the NAc decreases the acquisition of cocaine self-administration (Neumann et al., 2016). Moreover, transient inactivation of the PVT prevents cocaine seeking as well as cue-induced reinstatement of cocaine seeking (James et al., 2010; Matzeu et al., 2015a). These data suggest that PVT-to-NAc projections positively affect the locomotor activating and reinforcing effects of cocaine.

The finding that PVT D2R overexpression attenuates cocaine locomotor sensitization (Fig. 6) is in agreement with the above-mentioned lesion studies (Young and Deutch, 1998). Based on the dense projections to NAc shell, we hypothesize that D2R overexpression may inhibit cocaine locomotor sensitization by decreasing activity of the glutamatergic projections from the PVT to the NAc shell. Specifically, activation of presynaptic PVT D2Rs within the NAc might inhibit glutamatergic release by Gβγ-mediated inhibition of voltage-gated calcium channels or by activation of Kv1.2 potassium channels. Such mechanisms have been postulated for presynaptic D2 autoreceptor-mediated inhibition of transmitter release in dopaminergic neurons (Herlitze et al., 1996; Ikeda, 1996; Martel et al., 2011).

### D2R upregulation in the PVT does not affect fear conditioning

Surprisingly, D2R upregulation in the PVT did not affect fear conditioning. Lesions of the posterior PVT have been shown to inhibit cued fear expression (Li et al., 2014b) and silencing PVT projections to the central nucleus of the amygdala (CeA) impairs fear retrieval (Do-Monte et al., 2015). Furthermore, silencing projections from the PVT to the lateral CeA either during cued fear conditioning or during retrieval 24 h following fear conditioning decreases fear retrieval (Penzo et al., 2015). In addition, as discussed above, we also did not observe deficits in anxiety related behaviors or reward seeking as one might have expected.

One reason for the mild behavioral effects may be a possible ceiling effect whereby maximal inhibition of PVT activity by endogenous DA release is already achieved by wild-type D2R levels. Alternatively, the mild behavioral effects observed may be a result of the limited number of PVT neurons infected by the virus. Future studies examining the effect of knocking out D2R selectively in the PVT will determine if cocaine locomotor sensitization is bidirectionally modulated by PVT D2Rs.

However, there is one important difference between our study and the studies discussed above, which is that within our study, inhibition of the PVT is dependent on endogenous DA release during behavior, which then acts on excess D2Rs. DA release during fear conditioning or in the other unaffected behaviors may be insufficient to activate enough D2Rs to inhibit PVT neurons. In contrast, by blocking DAT, cocaine injections may result in sufficient levels of dopaminergic activation of PVT D2Rs to promote inhibition during cocaine sensitization. Since DAT levels are low in the PVT (Fig. 1*E*), we do not necessarily believe that the effects of cocaine are manifested by altering dopaminergic signaling at the level of PVT cell bodies. Instead, we hypothesize that cocaine may elicit its actions by increasing dopaminergic signaling at the level of D2R-expressing PVT terminals within the NAc, a region where DAT is heavily expressed. Future studies will address whether D2Rs on PVT terminals inhibit transmitter release as has been shown for striatal projection and dopaminergic neurons (Tecuapetla et al., 2009; Kohnomi et al., 2012; Ford, 2014; Dobbs et al., 2016). Currently the relative contribution of somatic versus terminal inhibition by D2Rs and how this affects cocaine sensitization is unknown and will need to be addressed in the future.

In conclusion, overexpression of D2R in the PVT attenuated cocaine locomotor sensitization. This finding suggests that D2R-mediated inhibition of PVT neurons modulates the sensitivity to cocaine. Since alterations in the thalamic dopaminergic system have been measured in cocaine addiction, our findings may also have implications for human drug use (Volkow et al., 1997; Volkow et al., 2005).

## Synthesis

Reviewing Editor: Karen Szumlinski, University of California at Santa Barbara Decisions are customarily a result of the Reviewing Editor and the peer reviewers coming together and discussing their recommendations until a consensus is reached. When revisions are invited, a fact-based synthesis statement explaining their decision and outlining what is needed to prepare a revision will be listed below. The following reviewer(s) agreed to reveal their identity: Jeffery Steketee.

I have consulted with the two expert reviewers regarding your submission, which was revised from its original version for submission to eNeuro. Reviewer 1 was a previous reviewer of your original report and found the revised manuscript much improved over the original. Reviewer 2 viewed your report for the first time and thus, raised additional concerns that we all agree, if addressed, would improve the report even further. Both reviewers and I agree that the results presented in your report provides important neuroanatomical characterization of the PVT and presents some limitations of the pseudorabies virus approach that are worthy of publication. On the other hand, all three of us agree that the behavioral results are limited and could be augmented by the inclusion of an additional experiment in which D2 receptors are knocked-down, or in the very least, a discussion regarding the interpretational limitation of approaching the research question using only an over-expression strategy (i.e., potential for ceiling effects) should be discussed. Further, both reviewers agree that the Introduction of the report requires elaboration with respect to our current knowledge regarding “non-striatal” dopamine neurocircuits. This elaboration should include discussion of the results of the study from Kirouac's group (Li et al. 2014, Front. Neuroanatomy), which suggests that dopamine innervation of PVT likely comes from hypothalmus or PAG. Further, both reviewers and I agree that the Discussion could be bolstered by inclusion of alternative mechanisms through which D2 receptors are modulated within the PVT. Below, please find additional specific concerns/comments/suggestions offered by the reviewers. As these issues are addressable, I have deemed a decision of Revise/Resubmit as most appropriate for your report.

Reviewer 1:

I have only a two minor comments that the authors should address.

1)The authors state that “the overall expression pattern of GFP matches well with the D2R mRNA expression pattern”. In my view, the authors would be well served to attempt to quantify the overlap, if possible. This will give readers a sense of certainty about the specificity of the Cre line and thus the data presented in the manuscript.

2) It is interesting, and important, that the authors identified heterogeneity of spiking patterns in D2R+ PVT cells. I take the authors' point that more recordings might be required to carry out statistical comparisons on these firing types, but the authors should acknowledge this heterogeneity with reference to the Yeoh 2014 paper.

Otherwise, the authors are to be congratulated on a nice manuscript.

Reviewer 2:

1) First paragraph of introduction: The authors suggest that striatal systems have been the predominant dopamine region investigated in neuropsychiatric and neurological disorders. However other regions have been investigated and should at least be mentioned for clarity.

2) Introduction: Most of the introduction was simply a synopsis of the results of the present report. This should be limited to a sentence or two at most.

3) Methods: For the sensitization studies, it was mentioned that time day was a factor (i.e. cohorts of animals tested in the morning or afternoon. However, it is not clear that this factor was included in the data analysis.

4) Methods: There is some redundancy between the Animals for Behavioral Testing and the Experimental Design and Statistical Analysis sections.

5) Results/Discussion: The data, as stated by the authors, indicates that there is no DAT staining (indicative of no dopamine innervation) in the PVT, yet there are D2 receptors. Yet later in the discussion, the author states that cocaine acts in part by blocking DAT in PVT. These statements contradict one another. The authors should discuss alternative mechanisms by which D2 receptors could be activated to influence PVT function and behaviors.
